# Targeting NKG2D ligands in glioblastoma with a bispecific T-cell engager is augmented with conventional therapy and enhances oncolytic virotherapy of glioma stem-like cells

**DOI:** 10.1136/jitc-2023-008460

**Published:** 2024-05-09

**Authors:** Richard Baugh, Hena Khalique, Emma Page, Janet Lei-Rossmann, Peter Kok-Ting Wan, Timothy Johanssen, Daniel Ebner, Olaf Ansorge, Leonard W Seymour

**Affiliations:** 1 Department of Oncology, University of Oxford, Oxford, UK; 2 Target Discovery Institute, University of Oxford Nuffield Department of Medicine, Oxford, UK; 3 Nuffield Department of Clinical Neurosciences, University of Oxford, Oxford, UK

**Keywords:** Oncolytic Virotherapy, Immunotherapy, Central Nervous System Neoplasms, Antibodies, Bispecific, Oncolytic Viruses

## Abstract

**Background:**

Glioblastoma (GBM) almost invariably becomes resistant towards conventional treatment of radiotherapy and temozolomide (TMZ) chemotherapy, partly due to subpopulations of intrinsically resistant glioma stem-like cells (GSC). The oncolytic herpes simplex virus-1 G207 is a promising approach for GBM virotherapy although its efficacy in patients with GBM is often limited. Natural killer group 2 member D ligands (NKG2DLs) are minimally expressed by healthy cells but are upregulated by the DNA damage response (DDR) and in malignant cells with chronic DDR signaling, resulting in innate immune activation.

**Methods:**

We have designed a bispecific T-cell engager (BiTE) capable of cross-linking CD3 on T cells with NKG2DL-expressing GBM cells. We then engineered the G207 virus to express the NKG2D BiTE and secrete it from infected cells. The efficacy of the free BiTE and BiTE delivered by G207 was evaluated in combination with conventional therapies in GBM cells and against patient-derived GSCs in the context of T-cell activation and target cell viability.

**Results:**

NKG2D BiTE-mediated cross-linking of GBM cells and T cells causes antigen-independent T-cell activation, pro-inflammatory cytokine release, and tumor cell death, thereby combining direct viral oncolysis with BiTE-mediated cytotoxicity. Surface NKG2DL expression was further elevated on GBM cells following pretreatment with sublethal doses of TMZ and radiation to induce the DDR, increasing sensitivity towards G207-NKG2D BiTE and achieving synergistic cytotoxicity. We also demonstrate a novel strategy for targeting GSCs that are non-permissive to G207 infection but remain sensitive to NKG2D BiTE.

**Conclusions:**

We propose a potential model for targeting GSCs in heterogeneous tumors, whereby differentiated GBM cells infected with G207-NKG2D BiTE produce NKG2D BiTE locally, directing T-cell cytotoxicity towards the GSC subpopulations in the tumor microenvironment.

**WHAT IS ALREADY KNOWN ON THIS TOPIC**

- Glioblastoma (GBM) is an invariably fatal adult brain tumor that commonly recurs in patients following conventional therapy, partly due to subpopulations of therapy-resistant glioma stem-like cells (GSCs). The oncolytic herpes simplex virus G207 has demonstrated potential as a glioma immunotherapy agent, but to date has been limited by GSCs. Bispecific T-cell engagers (BiTEs) can direct T-cell cytotoxicity towards target cells expressing target ligands of interest. BiTEs expressed from oncolytic viruses offer an attractive approach to deliver therapeutics locally within the tumor.

**WHAT THIS STUDY ADDS**

- Here, we arm the G207 virus with a BiTE targeting natural killer group 2 member D ligands expressed on the surface of GBM cells and GSCs and offer a novel combination strategy to eliminate therapy-resistant GSCs and in combination with conventional GBM therapy.

**HOW THIS STUDY MIGHT AFFECT RESEARCH, PRACTICE OR POLICY**

- This research provides a novel strategy for targeting populations of cells in the tumor microenvironment which are resistant to conventional therapy or oncolytic virotherapy by arming viruses with a BiTEs.

## Background

Glioblastoma (GBM), isocitrate dehydrogenase-wildtype central nervous system WHO grade 4, (*GBM*) is the most common primary brain tumor in adults and is characterized by being highly aggressive, vascularized, infiltrative, and heterogeneous.^1^ The current standard of care therapy involves debulking surgery followed by concomitant radiotherapy and temozolomide (TMZ).^2^ Despite this, tumors invariably recur, with a median patient survival of around 15 months.^2^ Glioma stem cells (GSCs) are subpopulations of cells capable of self-renewal and differentiation; considered to be drivers of resistance to conventional therapy, GSCs repopulate the tumor with resistant cells.^3^

Oncolytic viruses (OVs) have emerged as promising immunotherapeutics for cancer and involve the use of viruses capable of selectively replicating within and killing cancer cells while sparing healthy cells. Oncolytic herpes simplex viruses-1 (oHSVs) are enveloped double-stranded DNA viruses that are among the most clinically advanced OVs, including talimogene laherparepvec achieving Food and Drug Administration and European Medicines Agency approval for use against metastatic melanoma in 2015 in America,^4^ and DELYTACT which gained approval for the treatment of GBM in Japan in 2021.^5^ All oHSVs used to date are deleted in both copies of the γ34.5 neurovirulence gene,^6^ encoding infected cell protein (ICP)34.5, which enables cancer selectivity but imposes a significant attenuation of the viruses in clinical settings. The oHSV G207 is a γ34.5-deleted virus with an inactivated ICP6 gene which prevents expression of the viral ribonucleotide reductase, imposing further selectivity for proliferating cells.^7^ G207 has been clinically evaluated in GBM,^8^ demonstrating a good safety profile, evidence of viral replication and some promising responses.^9 10^ However, it has been revealed that GSCs were non-permissive to G207 by restricting the translation of viral true late (TL) proteins, while serum-cultured differentiated GBM cells remained permissive.^11^ Effectively targeting GSCs, therefore, is essential to improve the efficacy of oHSVs in GBM.

The natural killer group 2 member D (NKG2D) receptor and its ligands (NKG2DLs) are key components in the innate immune control of viral infections and cancers.^12^ NKG2D is an activatory receptor expressed by natural killer (NK) cells, γδ T cells, CD8^+^ T cells, and some CD4^+^ T cells, and can bind to eight different NKG2DLs, major histocompatibility complex (MHC)-class-I-polypeptide-related sequence (MIC) A and MICB, and UL16-binding protein 1–6.^13^

Surface expression of NKG2DLs has been widely reported on several cancer types, including gliomas,^14–16^ but is mostly absent in healthy tissues.^17^ Strategies targeting NKG2DLs for GBM therapy have been explored, including the use of human γδ T cells in vitro,^18^ or engineered γδ and NKG2D-chimeric antigen receptor (CAR)-T cells in vivo.^19 20^ Given the link between the DNA damage response (DDR) and NKG2DL expression,^21^ evidence has emerged that DNA damage induced by TMZ and radiation therapy further upregulates NKG2DL on GBM cells and can increase sensitivity to NK cell lysis.^22^ Bispecific T-cell engager (BiTEs) targeting murine^23^ and human^24^ NKG2DLs have previously been explored, however, these have not been evaluated in GBM or used in combination with OVs. Therefore, a BiTE targeting human NKG2DLs may be an effective strategy for eradicating GBM cells and may complement the direct cytotoxicity of the G207 virus. We deployed the NKG2D receptor itself as the NKG2DL-binding domain, as opposed to a mono-specific single chain variable fragment (scFv) used in conventional BiTE formats, enabling binding to all eight NKG2DLs. This BiTE was found to activate both CD4^+^ and CD8^+^ T cells to kill NKG2DL-expressing GBM cells and to synergize with sublethal pretreatment doses of TMZ and radiation which enhance NKG2DL expression. When encoded into the G207 virus, mediating the local expression of the BiTE by infected GBM cells, the NKG2D BiTE showed potent cytotoxicity towards GSCs despite the lack of direct oncolysis by the G207 itself. We propose arming oHSVs with an NKG2D BiTE as a novel strategy to mediate two distinct therapeutic effects against heterogenous GBM tumors, targeting cytotoxicity towards both GSCs and bulk differentiated GBM cells.

## Methods

### Cell lines and cell culture

U87, HEK293A and Vero cells (American Type Culture Collection (ATCC)) were cultured and maintained in Dulbecco’s Modified Eagle Medium (DMEM, Sigma-Aldrich, UK) supplemented with 10% (v/v) heat-inactivated fetal calf serum (FCS, (Gibco)) unless otherwise stated. Cells were cultured in an incubator at 37°C and 5% CO_2_. Cell lines were routinely tested for Mycoplasma (MycoAlert Mycoplasma Detection Kit, Lonza, UK). U87 GSCs and primary human GSCs were cultured in neurobasal medium (NBM, (Gibco, UK)) supplemented with B-27 Plus supplement (Thermo Fisher Scientific, UK), 20 ng/mL recombinant human epidermal growth factor (EGF) basic (PeproTech, UK), 20 ng/mL recombinant human fibroblast growth factor (FGF) basic (PeproTech, UK), 2 mM GlutaMAX-I (Gibco) and 5 mL Pen-Strep (Gibco).^25^ E57 mesenchymal GSCs were cultured either in DMEM/F12 (Sigma-Aldrich) supplemented with 10 ng/mL EGF, 10 ng/mL FGF, 10 µg/mL Cultrex 3-D Culture Matrix Laminin I (RnD systems, UK), 5 mL B-27 Plus supplement (Gibco), 2.5 mL N-2 supplement (Gibco), 5 mL MEM non-essential amino acids (Gibco), 1 mL β-mercaptoethanol (Gibco), 7.25 mL glucose (Sigma-Aldrich), 0.8 mL bovine serum albumin (Gibco) and 5 mL Pen-Strep (Invitrogen, UK) per 500 mL of media, as described previously^26^; or in advanced DMEM (AdMEM (Gibco)) supplemented with 5 mL B-27 Plus supplement (Gibco), 2.5 mL N-2 supplement (Gibco), 20 ng/mL EGF and 20 ng/mL FGF, 5 µg/mL heparin (Sigma-Aldrich). E57 cells were cultured on plates and flasks precoated in 10 µg/mL laminin. E57 cells were also cultured in DMEM with 10% FCS to promote a switch from stem-like to more terminally differentiated characteristics.

### NKG2D staining

To assess the expression of all NKG2DLs, a recombinant human NKG2D with human IgG Fc domain (rhNKG2D-Fc, (R&D systems)) fusion protein was used as a primary for staining. The rhNKG2D-Fc was biotinylated using a Biotin Conjugation Kit (Lightning-Link, (Abcam, UK)) according to the manufacturer’s instructions. As an isotype control, recombinant human IgG1 (rhIgG1-Fc, (R&D systems)) was also biotinylated. PE-conjugated streptavidin (Strep-PE, (Thermo Fisher Scientific)) was used as a secondary stain.

### Engineering and producing NKG2D and Control BiTE

**Figure 1:**
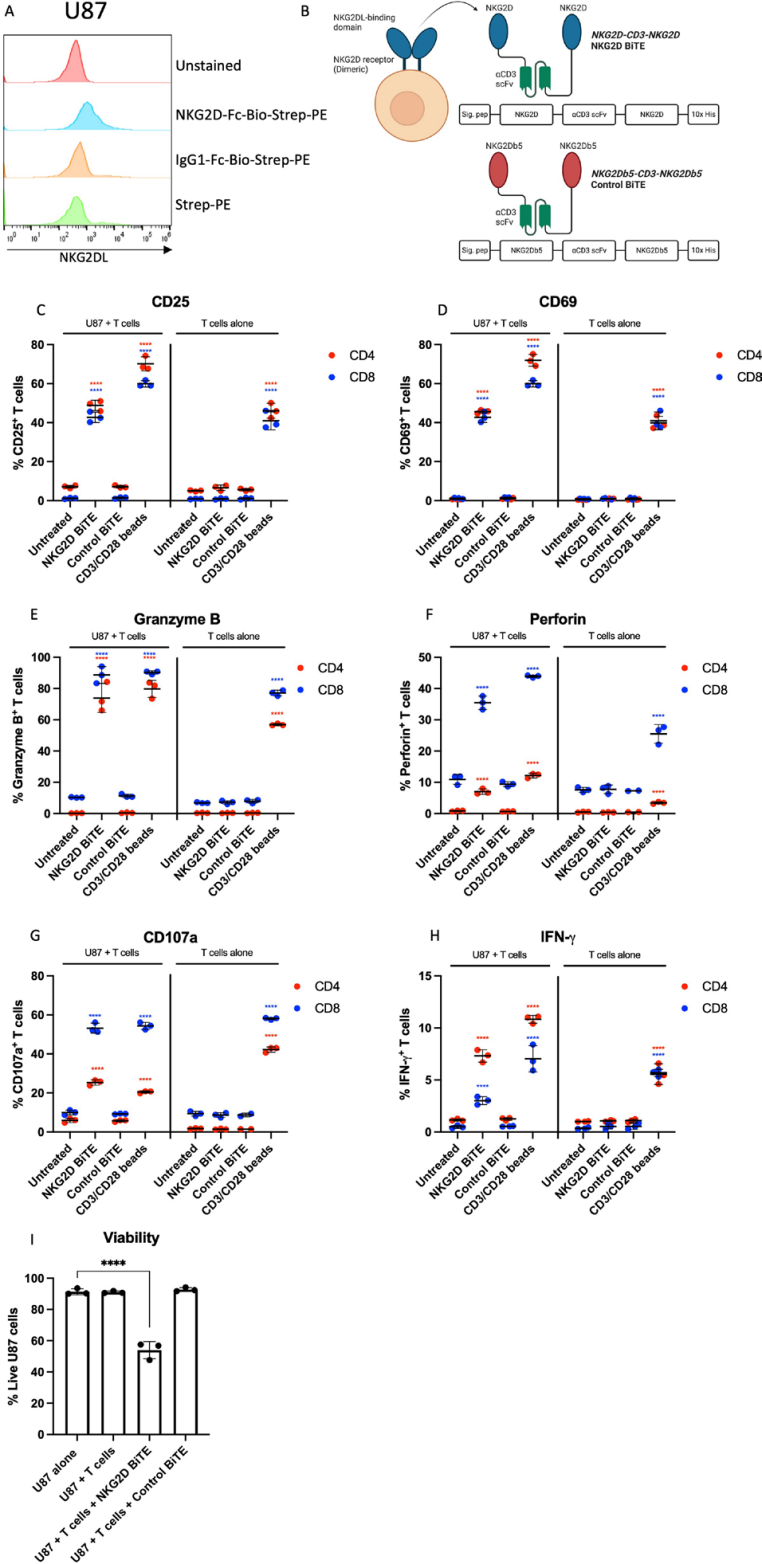
Characterization of NKG2D BiTE activity. (A) Representative flow cytometry histogram plots after human glioblastoma cells U87 were stained for total surface NKG2DLs using biotinylated recombinant human NKG2D-Fc and streptavidin-PE secondary (NKG2D-Fc-Bio-Strep-PE), biotinylated human IgG1 isotype (IgG1-Fc-Bio-Strep-PE), or streptavidin-PE secondary only (Strep-PE). (B) Schematic representation of NKG2D BiTE and Control BiTE. The ectodomain of human NKG2D receptor (amino acids 78–216) was used for the NKG2D BiTE. For the Control BiTE, amino acid residues in the stirrup loop (B5-B5’ loop) of NKG2D were mutated to abrogate ligand binding. NKG2D/NKG2Db5 ectodomains were linked to an anti-CD3ε single chain variable fragment (scFv) and a second NKG2D/NKG2Db5 ectodomain by flexible glycine-serine linkers. BiTEs contained N-terminal human immunoglobulin signal peptide and C-terminal decahistidine tag for mammalian secretion and protein detection. Diagram created with BioRender.com. (C) U87 cells were treated with 1 nM NKG2D BiTE, 1 nM Control BiTE, 1 μL CD3/CD28 Dynabeads or left untreated and co-cultured with peripheral blood mononuclear cell-derived T cells at an effector:target ratio of 5:1 for 72 hours before assessing expression of CD25, (D) CD69, (E) granzyme B, (F) perforin, (G) CD107a or lysosomal associated membrane protein-1 and (H) intracellular interferon (IFN-γ) on both CD4^+^ (red) and CD8^+^ (blue) T cells by flow cytometry. Data represents the mean percentage of positive T cells of triplicate repeats±SD. (I) U87 cell viability was assessed from the same experiments using a viability dye by flow cytometry. U87 cell viability was assessed either as untreated cells in monoculture, untreated co-cultures with T cells, co-cultures treated with 1 nM NKG2D BiTE or 1 nM Control BiTE. Data represents the mean percentage of live U87 cells of triplicate repeats±SD. Statistical significance was assessed by two-way ANOVA followed by Bonferroni post hoc analysis for (C)–(H) and an one-way ANOVA followed by Dunnett’s post hoc test for (I). Significance was assessed versus untreated cells within the relevant group or versus U87 cells alone in (I) (ns p>0.05, *p≤0.05, **p≤0.01, ***p≤0.001 and ****p≤0.0001). ANOVA, analysis of variance; BiTE, bispecific T-cell engager; NKG2D, natural killer group 2 member D; NKG2DLs, NKG2D ligands.

The ectodomain of the human NKG2D receptor (amino acids 78–216) was used for the NKG2D BiTE. For the Control BiTE, amino acid residues in the stirrup loop (B5-B5’ loop) of NKG2D were mutated from IIEMQ to RGGKR, turning the hydrophobic NKG2DL-binding pocket hydrophilic, thus preventing recognition and binding to NKG2DLs. Both NKG2D and mutated NKG2D (NKG2Db5) were constructed into the BiTE constructs by joining the ectodomains to an scFv recognizing CD3ε (clone L2K) and a second ectodomain (codon optimized to prevent DNA homology) via flexible glycine-serine linkers. An N-terminal immunoglobulin signal peptide and C-terminal decahistidine tag were included for mammalian secretion and detection (figure 1B). BiTE was produced by transfection of HEK293A cells with a plasmid encoding the BiTE using Lipofectamine 2000 (Invitrogen). Transfected cells were cultured for 72 hours before supernatants were harvested and concentrated as described previously.^27^ BiTE stocks were quantified by anti-His-tag ELISA (GenScript, UK).

### Generation of G207-NKG2D BiTE and G207-Control BiTE viruses

The HSVQuik system was used to engineer armed viruses into the MGH1 viral backbone (a kind gift from Ennio Chiocca, Harvard University, USA). The HSVQuik system uses the MGH1 viral backbone, which has the same deletions as G207 (infected cell protein (ICP)34.5Δ and ICP6Δ),^28^ and thus will be referred to as G207 henceforth. Viruses armed with NKG2D BiTE (G207-NKG2D BiTE) and Control BiTE (G207-Control BiTE) were generated by insertion of the BiTE cassette into the viral backbone in a bacterial artificial chromosome (BAC) as described previously.^28^ The parental G207 encodes in-frame green fluorescent protein (GFP) downstream of the truncated ICP6 coding sequence, while the BiTEs were placed under the control of the cytomegalovirus (CMV) promoter within the ICP6 region. The sequences of the BAC DNA were verified by Sanger sequencing (Genewiz, UK) before rescuing infectious viral particles via BAC excision.^28^ Individual viral plaques were isolated, characterized for BiTE expression by western blot and functional assays, amplified and concentrated by density ultracentrifugation.^29^ Infectious viral particles were titered using agar overlay plaque assay in Vero cells, and total viral particles were determined by Quant-iT Picogreen dsDNA assay as per the manufacturer’s instructions (Thermo Fisher Scientific). Virus genomes were calculated with the approximation of 0.5 ng of DNA equating to 3×10^6^ HSV-1 viral particles (vp).^30^ GBM cells were infected the day following seeding at a multiplicity of infection (MOI) of 0.1. Infections were carried out in Roswell Park Memorial Institute (RPMI) supplemented with 5% FCS and 10 mM 4-(2-hydroxyethyl)-1-piperazineethanesulfonic acid (HEPES) pH 7.3 unless otherwise stated.

### Viability assay

Cell viability of Vero cells was measured using CellTiter 96 AQueous One Solution Cell Proliferation Assay (MTS) kit (Promega, UK) according to the manufacturer’s instructions 72 hours after infection. Percentage viability was measured compared with uninfected controls.

### Processing of human peripheral blood mononuclear cells and T-cell isolation

Leukocyte cones from consenting healthy donors were acquired from the NHS Blood and Transplant Service (NHSBT; Oxford, UK). Peripheral blood mononuclear cells (PBMCs) were derived from whole blood by centrifugation using SepMate-50 columns (STEMCELL Technologies, UK) and Ficoll-Paque Plus (GE HealthCare, UK). T cells were isolated from the PBMC fraction with a Pan-T cell isolation kit (Miltenyi Biotec, UK). T cells were cultured in RPMI 1640 medium (Merck Life Sciences, UK) supplemented with 10% FCS, unless otherwise stated.

### Processing of clinical GBM tissue

Patient-derived GBM tissue was released from The Oxford Brain Bank obtained with informed consent from patients at the John Radcliffe Hospital, Oxford. GBM tumor tissue was obtained by cavitron ultrasonic surgical aspirator and was manually macerated using a scalpel blade before being mechanically and enzymatically dissociated into a single cell suspension using a Brain Tumor Dissociation Kit (Miltenyi Biotec) with C tubes (Miltenyi Biotec) and a GentleMACS Octo Dissociator (Miltenyi Biotec) according to the manufacturer’s instructions. Following dissociation, cell suspension was passed through a 70 µm cell strainer (Falcon, UK) and were then cultured in either 10% FCS RPMI or NBM at 37°C and 5% CO_2_.

### T-cell activation and target cell viability assays

Target cells were co-cultured with PBMC-derived T cells at an effector:target (E:T) ratio of 5:1 and were treated with NKG2D or Control BiTE in RPMI supplemented with 2% FCS. When viruses were used, target cells were infected for 4 hours prior to the addition of T cells in RPMI supplemented with 5% FCS and 10 mM HEPES pH 7.3. For co-cultures using GSCs or differentiated patient-derived GBM cells, co-cultures and viral infections were carried out in the same specific media. Co-cultures were incubated for 72–96 hours before harvesting cells for staining and flow cytometric analysis.

### Pretreatment with radiation and temozolomide

For pretreatment assays, cells were treated with either 50 µM TMZ (Thermo Fisher Scientific) or an equivalent volume of dimethyl sulfoxide (DMSO) vehicle control, exposed to 2 Gray (Gy) using a cesium-137 irradiator, or a combination of both TMZ and radiation. 48 hours following pretreatment, dead non-adherent cells were removed, live cells were seeded into plates to adhere overnight before functional assays as described above. Functional assays and assessments of ligand expression were performed the following day, after a total of 72 hours following pretreatment.

### Flow cytometry analysis

Prior to staining for flow cytometric analysis, adherent GBM cells were dissociated with non-enzymatic cell dissociation buffer (Gibco), while non-adherent T cells were collected from the cultures. Immune cells were treated with Fc receptor blocking agent (Miltenyi Biotec). For intracellular staining, cells were treated with GolgiStop and GolgiPlug (BD Biosciences, UK), according to the manufacturer’s instructions 5 hours before harvesting and staining. Following surface staining, cells were permeabilized with Perm/Wash (BioLegend) as per the manufacturer’s instructions. T cells were stained for with mouse anti-human CD4, CD8, CD25, CD69 CD107a, perforin, granzyme B (GzB) and interferon (IFN)-γ antibodies (BioLegend). For cell viability, cells were stained with LIVE/DEAD Fixable near-IR dead cell stain (Thermo Fisher Scientific). All staining was performed in MACS staining buffer, and cells were fixed using 10% neutral buffered formalin. Flow cytometry was performed on an Attune NxT Flow Cytometer (Thermo Fisher Scientific) and data was analyzed using FlowJo V.10.8.1 software (BD Biosciences, UK).

### Statistical analysis

Data processing and statistical analyses were performed using GraphPad Prism V.9.3.1. When comparing two data sets, a Student’s two-tailed t-test was used. Where more than two variables were being compared, a one-way analysis of variance (ANOVA) test was used, either with Tukey’s post hoc analysis, or with Dunnett’s post hoc analysis if one control condition was being compared. For grouped data sets with multiple variables, a two-way ANOVA test was used with Dunnett’s post hoc analysis compared with the control group. All data are presented alongside error bars indicating SD (±SD) unless otherwise specified. The significance levels used were ns p>0.05, *p≤0.05, **p≤0.01, ***p≤0.001 and ****p≤0.0001. All experiments were performed in triplicate unless otherwise stated.

## Results

### Generation and characterization of a BiTE targeting NKG2DLs

U87 cells demonstrated elevated expression of total surface NKG2DLs compared with staining controls (figure 1A) and were used as a model cell line to test the activity of the NKG2D BiTE in the context of GBM. The NKG2D BiTE was engineered using the NKG2D receptor ectodomain linked via a flexible glycine-serine linker to an anti-CD3 scFv and then to another NKG2D ectodomain (figure 1B). A Control BiTE was also constructed with an identical sequence except for amino acids in the NKG2D stirrup loop (B5-B5’ loop) were swapped from IIEMQ to RGGKR, preventing recognition and binding to NKG2DLs (figure 1B). Both BiTEs contained 10× Histidine residues for detection.

U87 cells were co-cultured with PBMC-derived T cells and treated with either NKG2D BiTE, Control BiTE, CD3/CD28 Dynabeads or left untreated before being harvested and stained for T-cell activation, effector molecules and cell viability for flow cytometric analysis. Treatment with NKG2D BiTE resulted in both CD4^+^ and CD8^+^ activation, with increases in CD25 and CD69 activation markers. Importantly this only occurred in the presence of target U87 cells (figure 1C and D), illustrating the need for the formation of an intercellular synapse for T-cell activation.

NKG2D BiTE induced cytotoxic effector molecule expression, with increased GzB, perforin and CD107a (figure 1E, F and G) only in the presence of target cells. Additionally, both CD4^+^ and CD8^+^ T cells had increased expression of IFN-γ (figure 1H). The Control BiTE did not cause any T-cell activation, demonstrating it was incapable of binding to NKG2DLs and that the formation of an immune synapse between T cells and target cells expressing NKG2DLs was required for activation. Only NKG2D BiTE caused a significant reduction in U87 cell viability, but not with Control BiTE or T cells alone (figure 1I).

### Combination therapy of radiation and temozolomide enhances NKG2D BiTE activity

**Figure 2:**
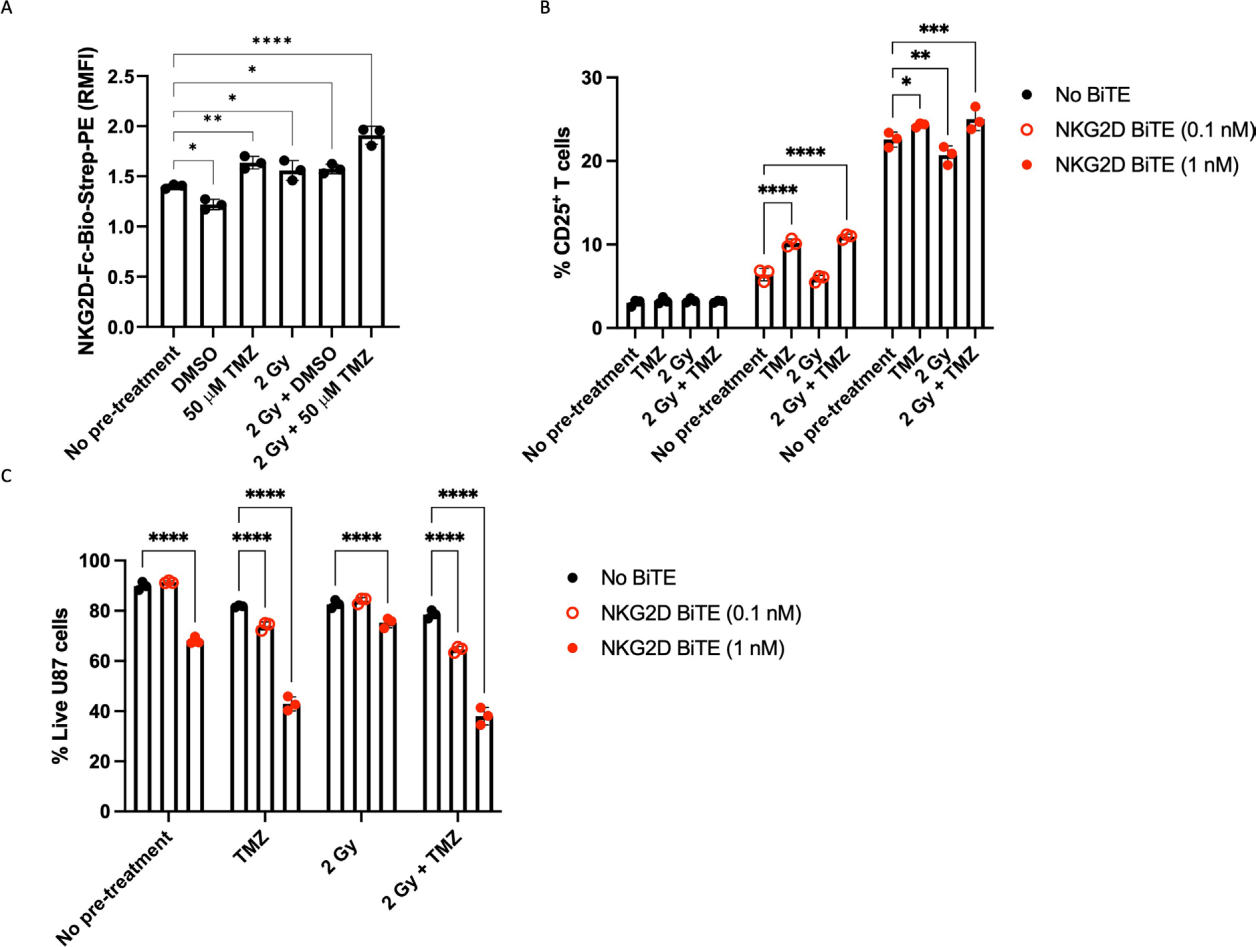
Pretreatment of U87 cells with a sublethal dose of TMZ and radiation increases NKG2DL expression and enhances NKG2D BiTE activity. (A) U87 cells were treated with single dose of 50 µM temozolomide (TMZ) or equivalent vehicle control (DMSO), or a single dose of 2 Gray (Gy) radiation either alone or in combination with 50 µM TMZ (2 Gy+TMZ) or vehicle control (2 Gy+DMSO), or left untreated (no pretreatment). 72 hours after pretreatment, surface NKG2DL expression was assessed by flow cytometry. Expression measured as relative mean fluorescence intensity (RMFI) relative to isotype from triplicate repeats±SD. Statistical significance was assessed by one-way ANOVA followed by Dunnett’s post hoc analysis. (B) U87 cells pretreated with TMZ, radiation or a combination were co-cultured with peripheral blood mononuclear cell-derived T cells and treated with 0.1 nM (hollow red circles) or 1 nM NKG2D BiTE (filled red circles), or no BiTE (black). After co-culturing for 72 hours, cells were harvested, stained and the percentage of CD25^+^ T cells and (C) live U87 cells was measured by flow cytometry. Data represents the mean percentage of positive or live cells from triplicate repeats±SD. Statistical significance was assessed by two-way ANOVA followed by Bonferroni post hoc analysis. Significance was assessed versus no pretreatment in (B) and versus no BiTE in (C) (ns p>0.05, *p≤0.05, **p≤0.01, ***p≤0.001 and ****p≤0.0001). ANOVA, analysis of variance; BiTE, bispecific T-cell engager; DMSO, dimethyl sulfoxide; NKG2D, natural killer group 2 member D; NKG2DLs, NKG2D ligands; RMFI, Relative median fluorescence intensity.

The expression of NKG2DLs is closely linked to the DDR; accordingly, we investigated how DNA damage induced by radiation and TMZ influenced NKG2DL expression and the resulting activity of the NKG2D BiTE. U87 cells were treated with a single dose of TMZ (50 µM), radiation (2 Gy), or a combination of both, doses chosen to be sublethal for U87 cells in monotherapy (online supplemental figure 1). Total surface NKG2DL expression was assessed by flow cytometry 72 hours after treatment and showed significant increases after each treatment relative to isotype-stained cells (figure 2A).

10.1136/jitc-2023-008460.supp1 Supplementary data

To investigate the effects of this ligand upregulation on the activity of NKG2D BiTE, U87 cells pretreated as above were co-cultured with PBMC-derived T cells and NKG2D BiTE using an optimal dose (1 nM) and a suboptimal dose (0.1 nM) (online supplemental figure 2) for a further 72 hours. T-cell activation was assessed by the expression of CD25 activation marker (figure 2B). In the absence of NKG2D BiTE no rise in CD25 expression was observed on T cells, while the low dose (0.1 nM) of BiTE caused a substantial increase in CD25 only when the U87 cells had been pretreated with TMZ or 2 Gy+TMZ, suggesting that pretreatment had increased the ability for low doses of the NKG2D BiTE to activate T cells. CD25 expression was also greatly increased at the higher BiTE dose, again significantly more when U87 cells that had been pretreated with TMZ or 2 Gy+TMZ. Intriguingly, pretreatment with 2 Gy alone caused a small but significant decrease in T-cell activation compared with no pretreatment (figure 2B).

10.1136/jitc-2023-008460.supp2 Supplementary data

The same experiment was used to compare U87 cell cytotoxicity of BiTE treatments in combination with pretreatments versus the pretreatment alone (figure 2C). Pretreatment without BiTE caused a slight fall in viability, while the 0.1 nM NKG2D BiTE treatment did not cause any cytotoxicity unless the U87 cells had been pretreated. The 1 nM NKG2D BiTE treatment mediated appreciable toxicity to U87 cells in co-cultures without pretreatment (figure 2C), although cytotoxicity was greater when U87 cells had been pretreated with TMZ and 2 Gy+TMZ pretreatment. Again, any potentiating effect of radiation alone on cytotoxicity of the NKG2D BiTE was very minor in comparison with the treatments involving TMZ. Pretreatments with DMSO had no increased effect on BiTE activity or target cell viability (online supplemental figure 3).

10.1136/jitc-2023-008460.supp3 Supplementary data

### Arming oHSV with NKG2D BiTE

**Figure 3:**
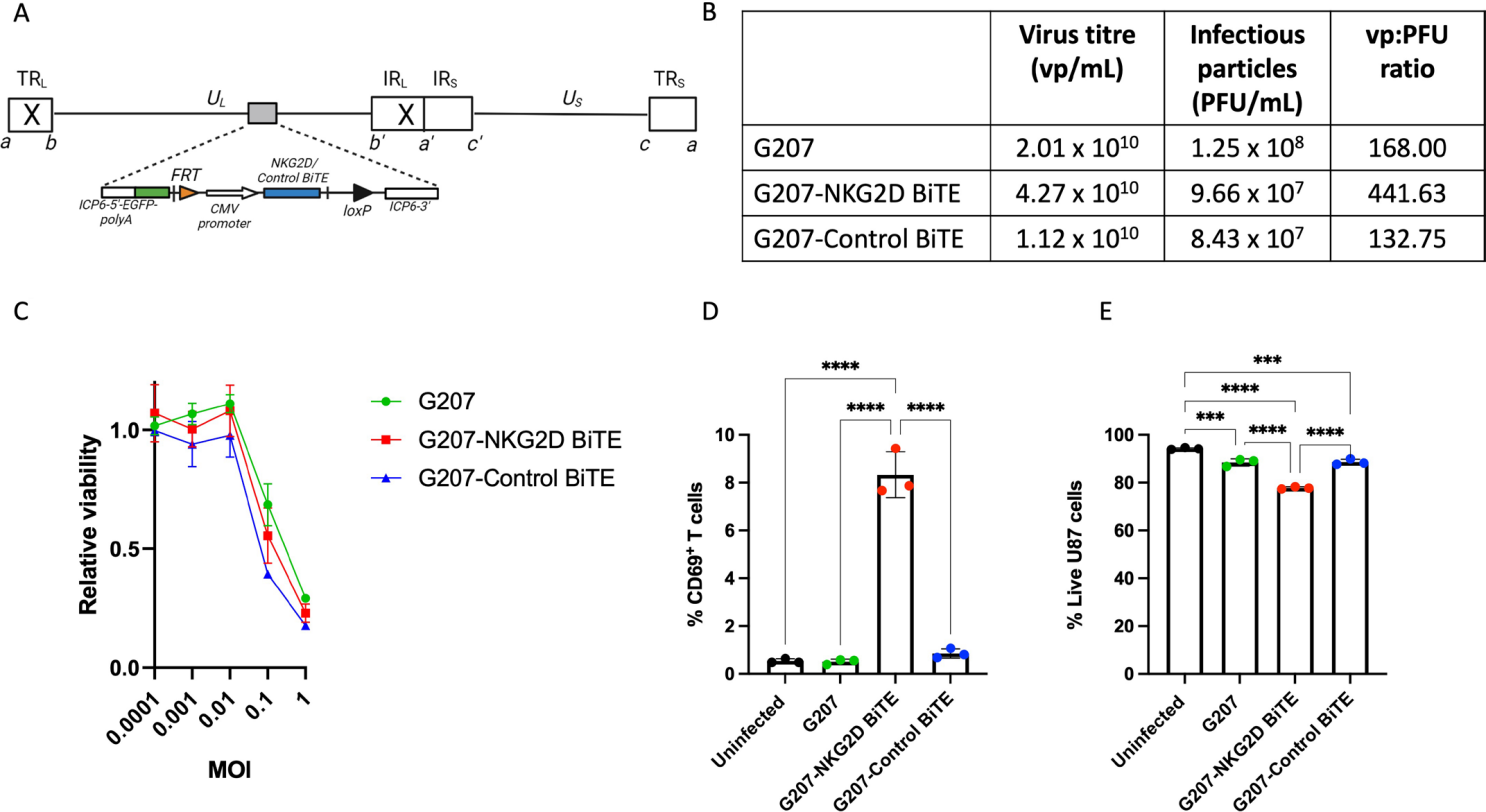
Generation and characterization of G207-NKG2D BiTE virus. (A) Schematic of G207-NKG2D BiTE virus genome. Linear overview of the viral genome structure, consisting of unique long (UL) and unique short (US) sequences, flanked by terminal inverted repeats: terminal repeat long (TRL; ab), terminal repeat short (TRS; ca), internal repeat long (IRL; b’a’) and internal repeat short (IRS; a’c’). Deletions of both copies of ICP34.5 are denoted by a cross. Insertion of green fluorescent protein (EGFP) in the ICP6 locus enables GFP expression under in place of ICP6. FRT and loxP recombination sites are included in the transgene to enable recombination into the viral genome. NKG2D BiTE or Control BiTE are inserted into the ICP6 locus under the control of the cytomegalovirus (CMV) promoter. Diagram adapted from Terada *et al*.^28^ (B) Table showing the quantitative assessments of G207, G207-NKG2D BiTE and G207-Control BiTE viruses. Virus titer of virus particles (vp)/mL was measured by PicoGreen assay. Infectious viral particles of plaque-forming units (PFU)/mL were measured by plaque agar assay. The ratio of virus particles: infectious vp (vp:PFU) was calculated by dividing the relative numbers. (C) Assessment of viral replicative activity. Vero cells were infected with G207 (green), G207-NKG2D BiTE (red) or G207-Control BiTE (blue) at MOI 0.0001, 0.001, 0.01, 0.1 and 1 for 72 hours before viability was measured by MTS assay. Relative viability is assessed compared with uninfected cells. Data represents mean relative viability of triplicate repeats±SD. (D) Characterization of BiTE expression by G207-NKG2D and G207-Control BiTE viruses. U87 cells were seeded and infected with parental G207 (green), G207-NKG2D BiTE (red) or G207-Control BiTE (blue) at a multiplicity of infection (MOI) of 0.1 or left uninfected (black). 4 hours after infection, peripheral blood mononuclear cell-derived T cells were added at an effector:target ratio of 5:1. After co-culturing for 72 hours, cells were harvested, stained and the percentage of CD69^+^ T cells was measured by flow cytometry. (E) The percentage of live U87 cells was assessed using a flow cytometric viability dye. Data represents the mean percentage of positive cells from triplicate repeats±SD. Statistical significance was assessed by one-way analysis of variance followed by Tukey’s post hoc test for (D) and (E). (ns p>0.05, *p≤0.05, **p≤0.01, ***p≤0.001 and ****p≤0.0001). BiTE, bispecific T-cell engager; NKG2D, natural killer group 2 member D.

In order to achieve local expression of the NKG2D BiTE in situ from U87 cells, the NKG2D BiTE was encoded into the G207-like backbone (MGH1), under the control of the CMV promoter (figure 3A). G207-NKG2D BiTE and G207-Control BiTE viruses were isolated, sequenced, concentrated, purified and characterized. They showed similar titers of total and infectious vp compared with parental (empty) oHSV (figure 3B). To assess whether insertion of the BiTE transgenes had affected the viral fitness and oncolytic activity, the BiTE-armed and parental G207 viruses were titrated on Vero cells (figure 3C) and showed that the armed viruses retained similar cytolytic activity as parental G207.

To assess BiTE function from the armed viruses, U87 cells were infected with G207, G207-NKG2D BiTE and G207-Control BiTE (MOI, 0.1) and co-cultured with PBMC-derived T cells for 72 hours. Flow cytometry of the T-cell activation marker CD69 (figure 3D) revealed a specific and significant increase in T-cell activation only in the presence of U87 cells infected with G207-NKG2D BiTE, demonstrating functional NKG2D BiTE was being produced and secreted into the medium. NKG2D BiTE secretion was further confirmed as conditioned media from U87 and Vero cells infected with G207-NKG2D BiTE triggered T-cell activation against fresh, uninfected U87 cells, whereas conditioned media from G207-Control BiTE did not (online supplemental figure 4). The lack of any T-cell activation in the presence of U87 cells infected with parental oHSV indicates that there was no immediate response towards the oHSV itself, and that T-cell activation was NKG2D BiTE-specific. U87 cell viability was also assessed (figure 3E) and showed a minor decrease in viability with parental G207 and G207-Control BiTE, reflecting the direct oncolytic activity of the virus itself, while G207-NKG2D BiTE virus caused a significantly greater viability decrease, reflecting the combination of the oncolytic virus activity with T cell-mediated killing induced by the NKG2D BiTE.

10.1136/jitc-2023-008460.supp4 Supplementary data

### Combination therapy of radiation and temozolomide enhances G207-NKG2D BiTE oncolytic virotherapy

To assess the anti-glioma activity of the armed viral construct, U87 cells were pretreated as above, before being infected with a low MOI (0.1) of G207-NKG2D BiTE virus, G207-Control BiTE virus or parental G207. Low MOI was used to accentuate the cytotoxicity of the NKG2D BiTE activity rather than direct oncolysis.

**Figure 4:**
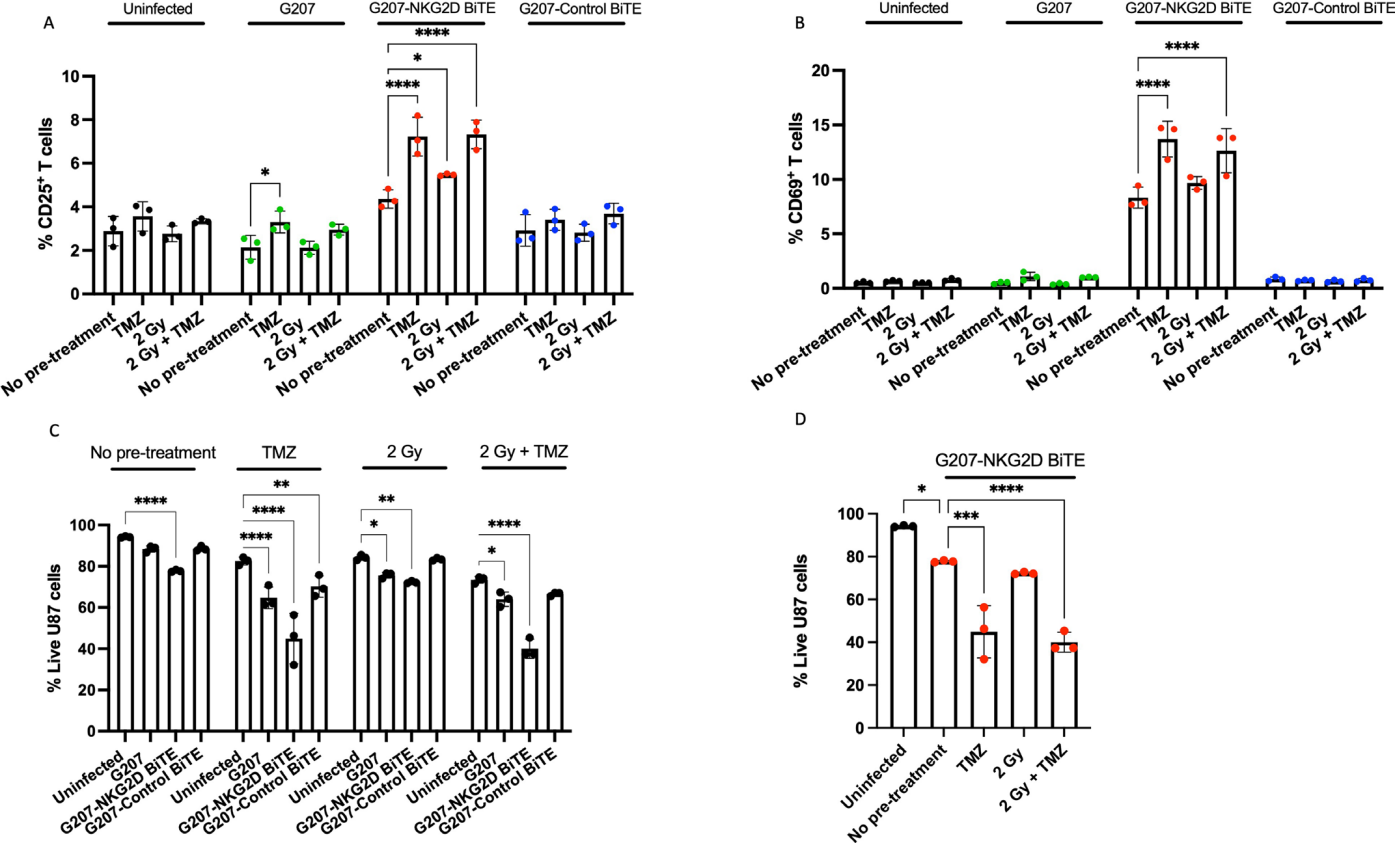
Sublethal pretreatment of TMZ and radiation enhances G207-NKG2D BiTE killing of U87 cells. (A) U87 cells were treated with 50 µM temozolomide (TMZ), a single dose of 2 Gray (Gy) radiation either alone (2 Gy) or in combination with 50 µM TMZ (2 Gy+TMZ) or left untreated (no pretreatment). 72 hours after pretreatment, U87 cells were seeded and infected with parental G207 (green), G207-NKG2D BiTE (red) or G207-Control BiTE (blue) at a multiplicity of infection of 0.1 or left uninfected (black). 4 hours after infection, peripheral blood mononuclear cell-derived T cells were added at an effector:target ratio of 5:1. After co-culturing for 72 hours, cells were harvested, stained and the percentage of CD25^+^, (B) CD69^+^ T cells and (C) live U87 cells was measured by flow cytometry. (D) An alternate representation of the data from (C). Data represents the mean percentage of positive cells from triplicate repeats±SD. Statistical significance was assessed by two-way ANOVA followed by Bonferroni post hoc analysis for (A) (B) and (C) and by one-way ANOVA followed by Tukey’s post hoc test for (D). Significance was assessed versus uninfected cells within the relevant groups for (A) (B) and (C) and versus no pretreatment for (D) (ns p>0.05, *p≤0.05, **p≤0.01, ***p≤0.001 and ****p≤0.0001). ANOVA, analysis of variance; BiTE, bispecific T-cell engager; NKG2D, natural killer group 2 member D.

As observed previously, T cells were not activated (assessed by expression of CD25 and CD69) in co-cultures with uninfected U87 cells or cells infected with control viruses, independent of any pretreatments (figure 4A and B). However, pretreatment with either TMZ or radiation (or both) significantly increased the T-cell activation achieved by the G207-NKG2D BiTE virus. Similarly, the viability of U87 cells was largely unaffected by pretreatment alone or infection with control viruses (figure 4C). Infection with G207-NKG2D BiTE virus alone resulted in significant cytotoxicity to U87 cells, and this was further enhanced in combination with TMZ or 2 Gy+TMZ pretreatments (figure 4D). Infection with parental G207 and G207-Control BiTE also caused significantly greater toxicity when combined with TMZ or 2 Gy+TMZ pretreatments (figure 4C). Together, these results suggest that the increased surface NKG2DL expression following pretreatment, particularly with TMZ, resulted in increased BiTE-mediated T-cell activation and enhanced NKG2D BiTE-mediated cytotoxicity, killing significantly more U87 cells than conventional therapy or parental G207.

### NKG2D BiTE targets both oHSV-permissive GBM cells and non-permissive GSCs

**Figure 5:**
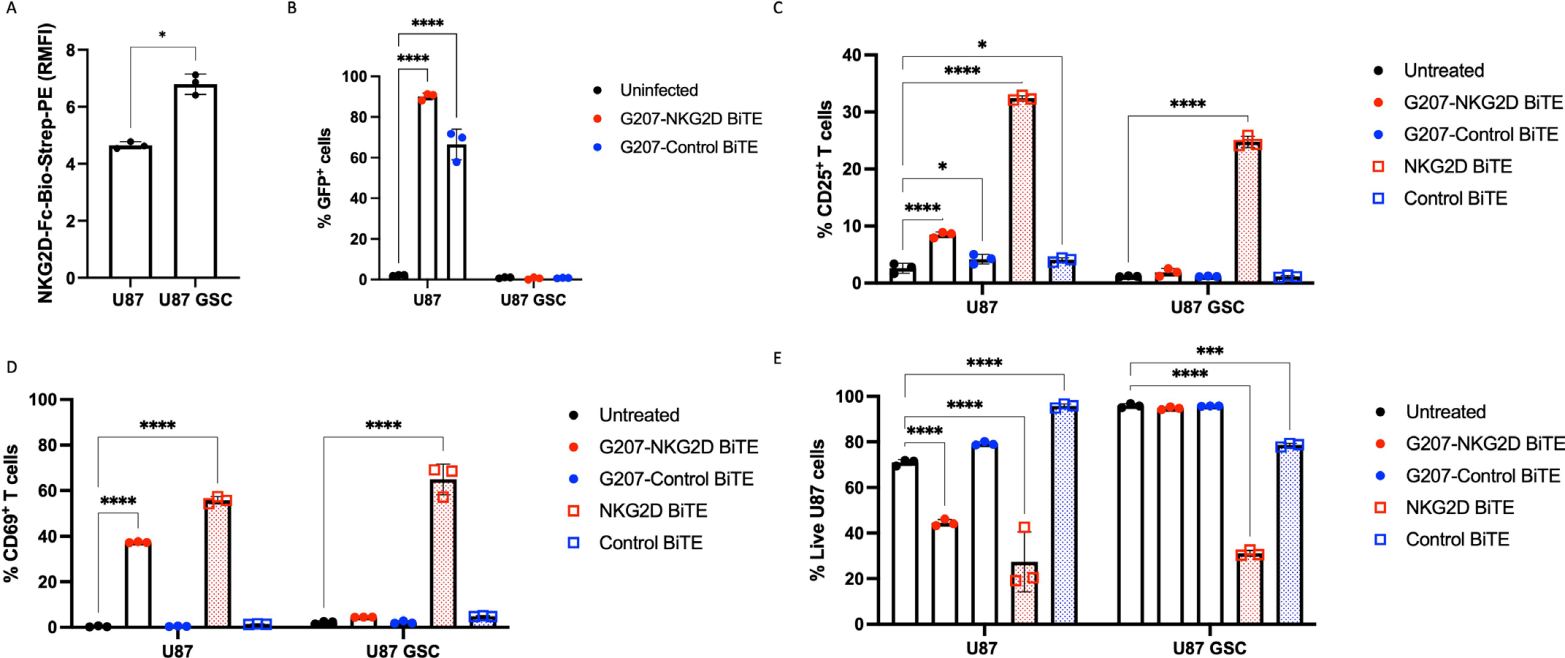
U87 GSCs remain sensitive to NKG2D BiTE despite being non-permissive to G207 virus. (A) U87 cells were cultured either as differentiated cells or as GSC-like cells and their surface NKG2DL expression was assessed by flow cytometry. Expression measured as relative mean fluorescence intensity (RMFI) relative to isotype from triplicate repeats±SD. Statistical significance was assessed by Student’s two-tailed t-test. (B) Differentiated U87 and U87 GSCs were infected with G207-NKG2D BiTE (red) or G207-Control BiTE (blue) at a multiplicity of infection (MOI) of 0.1 or left uninfected (black) for 96 hours before assessing virus infectivity by green fluorescent protein expression by flow cytometry. (C) Differentiated U87 and U87 GSCs were infected with G207-NKG2D BiTE (red circles) or G207-Control BiTE (blue circles) at an MOI of 0.1, or treated with 1 nM free NKG2D BiTE (red squares) or Control BiTE (blue squares) and co-cultured with peripheral blood mononuclear cell-derived T cells at an effector:target ratio of 5:1. After co-culturing for 96 hours, cells were harvested, stained and the percentage of CD25^+^ and (D) CD69^+^ T cells and (E) live U87 cells was assessed by flow cytometry. Data represents the mean percentage of positive cells from triplicate repeats±SD. Statistical significance was assessed by two-way analysis of variance followed by Bonferroni post hoc analysis. Significance was assessed versus uninfected cells for (B) and untreated cells within the relevant cell type for (C) (D) and (E). (ns p>0.05, *p≤0.05, **p≤0.01, ***p≤0.001 and ****p≤0.0001). BiTE, bispecific T-cell engager; GSCs, glioma stem cells; NKG2D, natural killer group 2 member D; NKG2DLs, NKG2D ligands.

Patient-derived GSCs have previously been shown to be non-permissive to G207 oHSV infection, unlike differentiated serum-cultured GBM cells, due to an inhibition of TL protein translation,^11^ and present an obstacle for G207 oncolytic virotherapy for GBM. Therefore, we sought to investigate whether GSCs could be targeted by the NKG2D BiTE. We initially used U87 cells cultured as differentiated GBM cells in normal serum conditions or conditions to promote stem-like features (hereby referred to as U87 GSCs) as a cell line model to investigate NKG2D BiTE activity.^25^ Assessment of relative surface NKG2DL expression revealed that U87 GSCs had significantly increased NKG2DLs compared with differentiated U87 cells (figure 5A). The G207-like virus (MGH1) has an insertion of GFP into the ICP6 locus, enabling the detection of infected cells by flow cytometry. Differentiated U87 cells and GSCs were treated with oHSV viruses and only differentiated U87 cells demonstrated a significant increase in GFP^+^ cells after infection (96 hours) with G207-NKG2D BiTE or G207-Control BiTE virus (figure 5B). Differentiated cells and GSCs were then either infected with G207-NKG2D BiTE or G207-Control BiTE, or treated with exogenously added free NKG2D/Control BiTE, and co-cultured with PBMC-derived T cells. Differentiated U87 cells induced a strong T-cell activation following the addition of free NKG2D BiTE, and when infected with G207-NKG2D BiTE virus they released sufficient NKG2D BiTE into the culture supernatants also to trigger T-cell activation, although at a lower level under these conditions (figure 5C and D). However, U87 GSCs infected with G207-NKG2D BiTE did not induce any T-cell activation, likely due to a lack of NKG2D BiTE production from these non-permissive GSCs. Despite this, U87 GSCs treated with free NKG2D BiTE still induced a strong T-cell activation (figure 5C and D). Reflecting this pattern of T-cell activation, only U87 GSCs treated with free NKG2D BiTE had decreased viability but not when infected with G207-NKG2D BiTE. Meanwhile, differentiated U87 cell viability was sensitive to both G207-NKG2D BiTE virus and free NKG2D BiTE. These results indicate that despite being non-permissive to oHSV infection, the U87 GSCs can still be targeted and killed by the NKG2D BiTE.

### NKG2D BiTE targets primary GBM cells and GSCs

**Figure 6:**
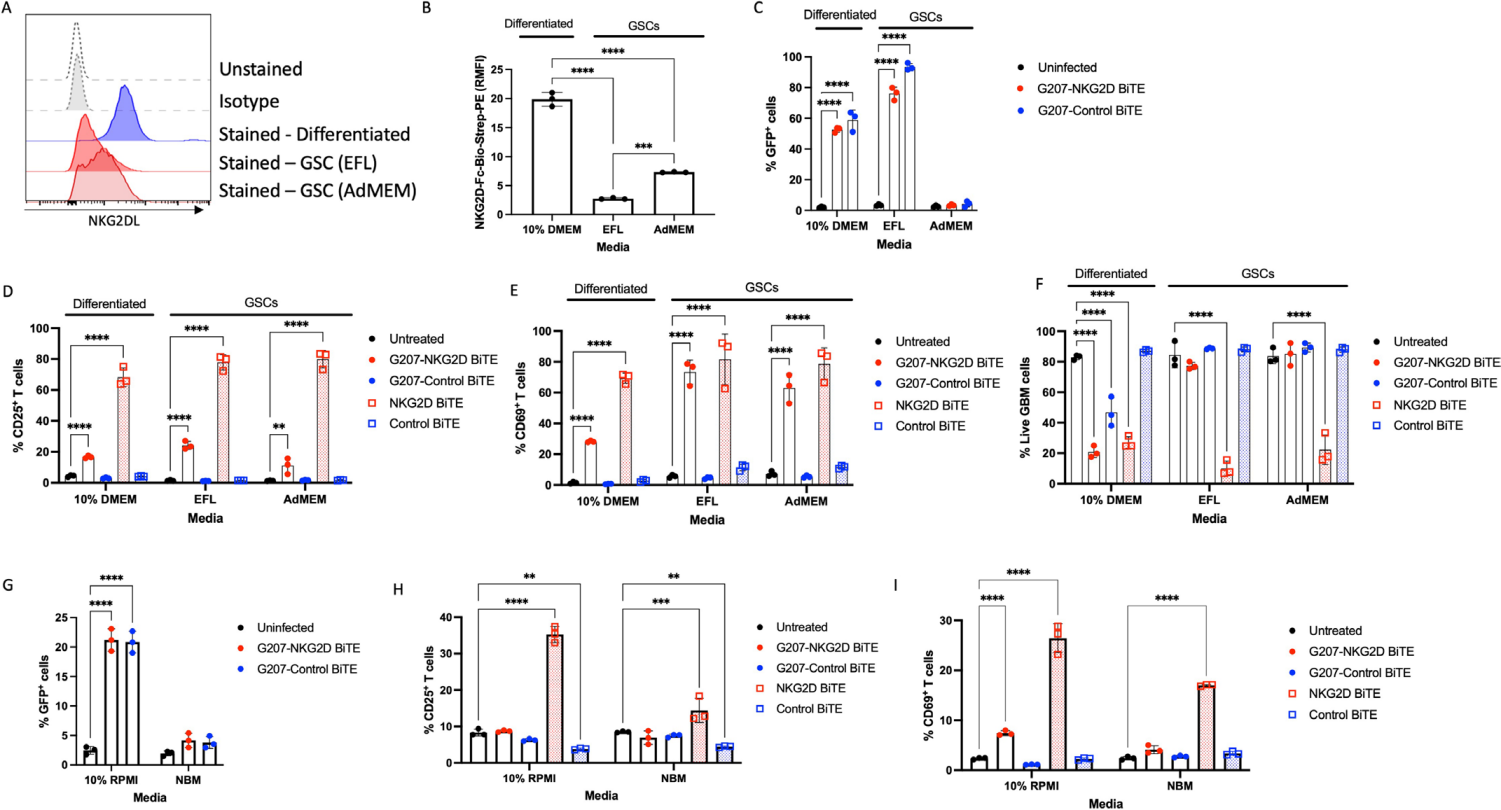
Patient-derived GSCs and primary GSCs can be targeted by NKG2D BiTE. Patient-derived glioma stem-like cells (GSCs), E57 cells, were cultured in DMEM supplemented with 10% fetal calf serum to promote differentiation or cultured in two different medias to promote GSC-like phenotype (EFL and AdMEM). (A) E57 cells were stained for total surface NKG2DLs using biotinylated recombinant human NKG2D-Fc and streptavidin-PE secondary. The figure shows representative flow cytometry histogram plots of E57 GSCs unstained (dotted line), isotype stained GSCs (gray dotted line), and stained with NKG2D-Fc-streptavidin-PE when cultured as differentiated cells (blue) or GSC-like cells (red). (B) Relative median fluorescent intensity (RMFI) of differentiated E57 cells and GSCs stained for total NKG2DLs compared with isotype. (C) E57 cells cultured in 10% DMEM, EFL or AdMEM were infected with G207-NKG2D BiTE (red) or G207-Control BiTE (blue) at a multiplicity of infection (MOI) of 0.1 or left uninfected (black) for 96 hours before assessing virus infectivity by GFP expression by flow cytometry. (D) E57 cells in each media were infected with G207-NKG2D BiTE (red circles) or G207-Control BiTE (blue circles) at an MOI of 0.1, or treated with 1 nM free NKG2D BiTE (red squares) or Control BiTE (blue squares) and co-cultured with PBMC-derived T cells at an effector:target ratio (E:T) of 5:1. After co-culturing for 96 hours, cells were harvested, stained and the percentage of CD25^+^ and (E) CD69^+^ T cells, and (F) E57 cell viability was assessed by flow cytometry. (G) Human GBM tissue obtained by cavitron ultrasonic surgical aspirator from one patient was dissociated and cultured in either neurobasal medium (NBM) supplemented with recombinant human fibroblast growth factor (FGF) and epidermal growth factor (EGF) to encourage a GSC phenotype, or in RPMI containing 10% FCS to encourage a differentiated phenotype. Primary GBM cells were infected with G207-NKG2D BiTE (red) or G207-Control BiTE (blue) at an MOI of 0.1 or left uninfected (black) for 96 hours before assessing virus infectivity by GFP expression by flow cytometry. (H) E57 cells in each media were infected with G207-NKG2D BiTE (red circles) or G207-Control BiTE (blue circles) at an MOI of 0.1, or treated with 1 nM free NKG2D BiTE (red squares) or Control BiTE (blue squares) and co-cultured with PBMC-derived T cells at an E:T ratio of 5:1. After co-culturing for 96 hours, cells were harvested, stained and the percentage of CD25^+^ and (I) CD69^+^ T cells was assessed by flow cytometry. Data represents the mean percentage of positive cells or RFMI from triplicate repeats±SD. Statistical significance was assessed by two-way ANOVA followed by Bonferroni post hoc analysis, or one way ANOVA followed by Tukey’s post hoc test for (B). Significance was assessed versus uninfected cells for (C) and (G) or untreated cells within the relevant cell type for (D) (E) (F) (H) and (I). (ns p>0.05, *p≤0.05, **p≤0.01, ***p≤0.001 and ****p≤0.0001). AdMEM, advanced DMEM; ANOVA, analysis of variance; BiTE, bispecific T-cell engager; DMEM, Dulbecco’s Modified Eagle Medium; EFL, EGF, FGF, laminin; GBM, glioblastoma; GFP, green fluorescent protein; NKG2D, natural killer group 2 member D; NKG2DLs, NKG2D ligands; PBMC, peripheral blood mononuclear cell; RMFI, Relative median fluorescence intensity; RPMI, Roswell Park Memorial Institute.

We next sought to confirm the findings that the NKG2D BiTE could target GSCs by using patient-derived GSCs and primary glioma tissue. Patient-derived mesenchymal E57 GSCs^26^ were cultured either in a normal medium containing serum to promote differentiation (DMEM with 10% FCS) or in one of two different medias containing growth factors and supplements to promote stem cell-like phenotypes (EGF, FGF, laminin (EFL) and AdMEM). Staining for surface NKG2DL expression revealed that all phenotypes expressed high levels of NKG2DLs, with serum-cultured E57 cells having the highest expression (figure 6A and B).

E57 cells were then infected with either G207-NKG2D BiTE or G207-Control BiTE virus in each respective media and virus infection was measured by GFP expression by flow cytometry (figure 6C). While differentiated E57 cells were highly permissive to G207, E57 GSCs cultured in AdMEM showed low permissivity to G207. Surprisingly, however, E57 GSCs cultured in EFL demonstrated high levels of GFP^+^ cells. E57 cells were then infected with G207-NKG2D BiTE or G207-Control BiTE, or treated with free NKG2D or Control BiTE, and co-cultured with PBMC-derived T cells, before assessing T-cell activation and cell viability (figure 6D, E and F respectively). Serum-cultured E57 cells triggered T-cell activation and a reduction in viability in response to both G207-NKG2D BiTE virus and free NKG2D BiTE. E57 GSCs cultured in either stem cell media also resulted in T-cell activation (figure 6D and E), but only free NKG2D BiTE caused a reduction in viability and not the G207-NKG2D BiTE virus (figure 6F). Together these results indicate that the patient-derived E57 GSCs were highly sensitive to NKG2D BiTE, but not oHSV. Interestingly, E57 GSCs cultured in an EFL medium resulted in high numbers of GFP^+^ cells when infected with either G207-NKG2D BiTE or Control BiTE virus, but no direct viral-mediated oncolytic activity was observed. In contrast, E57 GSCs cultured in AdMEM had no significant increase in GFP^+^ cells compared with uninfected cells, suggesting a lower permissivity. Despite these differences in GFP expression with G207 infection, both GSC types triggered T-cell activation when infected with G207-NKG2D BiTE virus, indicating that the NKG2D BiTE was still being produced from infected cells. This may perhaps suggest that the two different growth conditions to promote GSC phenotypes allow the G207-NKG2D BiTE virus to infect the GSCs and progress to different stages in the viral life cycle. Although neither GSC underwent direct oncolysis with G207-NKG2D BiTE, infected cells remained able to produce BiTE.

To further confirm that the NKG2D BiTE could target GSCs, cells obtained from primary human GBM surgical aspirate were dissociated and cultured either in a normal medium containing serum (RPMI with 10% FCS) or GSC-promoting conditions (NBM) and infected with G207-NKG2D BiTE or G207-Control BiTE virus and co-cultured with healthy donor PBMC-derived T cells. Differentiated primary GBM cells were permissive to G207, with distinct populations of GFP^+^ cells detected by flow cytometry (figure 6G), but not when cultured in GSC-promoting conditions. Both serum-cultured and NBM-cultured primary GBM cells could activate T cells when treated with free NKG2D BiTE (figure 6H), but only serum-cultured primary GBM cells supported enough viral infection for a significant increase in CD69 activation marker to be detected when infected with G207-NKG2D BiTE virus (figure 6I).

## Discussion

GBM remains a devastating disease, presenting one of the worst prognoses of all cancer types. After surgery, TMZ and radiation have emerged as the most effective treatment strategies although their efficacy is often only marginal. oHSVs are gaining traction as a new treatment strategy, with the first agent, DELYTACT, now licensed in Japan for the treatment of GBM.^5^ The anticancer activity of OVs can often be augmented and diversified by arming them to produce therapeutics, such as BiTEs, selectively within the tumor microenvironment. Accordingly, we hypothesized that mechanistic synergy might be achieved by arming an oHSV with a BiTE that targets ligands that are induced on GBM cells that survive TMZ and radiotherapy, such as NKG2DLs.^19 22 31^

Combining TMZ and radiation with NKG2DL-targeting immunotherapies has already been studied in GBM, revealing a pattern of increased antitumor activity due to increased NKG2DL expression. Weiss *et al*, demonstrated that TMZ and radiation enhanced the immunogenicity in a murine syngeneic orthotopic GBM model in an NKG2D-dependent manner.^22^ Lamb *et al*, observed increased survival in immunodeficient mice with orthotopic patient-derived xenografts administered with TMZ-resistant γδ T cells when used in combination with TMZ.^32^ Additionally, Weiss *et al* demonstrated synergistic activity when using an NKG2D-CAR-T cell in combination with a sublethal dose of radiation in a murine syngeneic glioma model, owing to the increased NKG2DL expression following radiation.^20^ Furthermore, U87 cells cultured to promote stem-like features demonstrated increased sensitivity towards NK-mediated killing due to increased NKG2DL expression.^25^ However, administering NKG2D-targeting approaches systemically could lead to on-target, off-tumor reactions if there were other ‘stressed’ cells in the body. These potential issues may be less likely to impact the activity of the G207-NKG2D BiTE virus, as expression of the NKG2D BiTE will be restricted to the tumor due to virus selectivity.

Interestingly, pretreatment of GBM cells with TMZ and radiation in co-culture with T cells did not induce physiological NKG2D receptor-dependent T-cell activation, as T cells only became activated if NKG2D BiTE was added. This suggests that rerouting NKG2DL signaling through CD3 (using a BiTE) is better able to activate T cells than by NKG2D receptor-mediated activation. Similarly, although NK cells are the main mediators of physiological NKG2DL-induced responses, there are relatively few tumor-infiltrating NK cells in GBM compared with other immune cells,^33^ and transforming growth factor-β expressed by GBM cells has been shown to downregulate NK cell expression of NKG2D receptor.^16^ It follows that redirecting NKG2DLs to activate a broad spectrum of tumor infiltrating T cells via CD3 activation may encourage more powerful immune responses against NKG2DL-expressing GBM cells.

GBM is generally considered to have relatively poor infiltration of tumor-infiltrating lymphocytes (TILs),^34^ which could limit the efficacy of the NKG2D BiTE. oHSVs, including G207, have demonstrated increased TILs and improved pro-inflammatory signatures following viral administration in patients with GBM,^35 36^ with observations made in post-treatment biopsies taken within a week following oHSV treatment,^35 37^ but even as little as 2 days post-G207 treatment.^9^ Given the kinetics of NKG2D BiTE activation following infection of GBM cells with G207-NKG2D BiTE virus takes around 72 hours, this timing of TIL infiltration could coincide with the accumulation of NKG2D BiTE to therapeutically effective doses. G207-NKG2D BiTE virus could also further be enhanced with arming to express chemokines to recruit more TILs. Furthermore, since the NKG2D BiTE binds to CD3, it can induce T-cell receptor-independent activation of both CD4^+^ and CD8^+^ T cells. Given the observations that MHC-I expression is frequently downregulated in GBM^38^ to evade antigen-specific T-cell killing, an MHC-independent strategy involving the NKG2D BiTE may repurpose many of the TILs present in the tumor microenvironment (TME). Finally, BiTEs are known to induce T-cell proliferation following activation,^27 39^ and so NKG2D BiTE-mediated activation may trigger TIL expansion in the GBM TME, increasing the number of effector cells for BiTE therapy. All these factors support the combination of OVs and BiTEs to augment immunotherapy, and the choice of NKG2DLs as a target provides a potentially pan-cancer stress-induced vulnerability, largely independent of any specific genetic mutations.

The expression of NKG2DLs has widely been reported on several cancer types, not just GBM.^15 31^ However, surface NKG2DL expression on healthy cells is mostly absent,^17^ although intracellular MICA and MICB have been reported in normal tissues.^40^ Given surface NKG2DL expression is mechanistically tied to various general phenotypes associated with cell stress and malignancy, such as the DDR and excessive proliferation,^21 41 42^ it may be difficult for cancer cells to avoid these general cancer-associated phenotypes. Cancers have evolved several means to evade the subsequent immune response to NKG2DL expression^12^ by dysregulating the NKG2D response further downstream, such as ligand shedding^43 44^ or immune subversion.^16 45^ Despite these downstream immune evasion tactics, the root of the cause for NKG2DL expression remains: the cancer phenotype itself. Perhaps with additional augmentation to prevent these immune evasion tactics, such as inhibiting NKG2DL shedding with matrix metalloprotease inhibitors^46^ or antibodies that occlude the enzymatic cleavage site,^47^ NKG2DLs may serve as attractive pan-cancer antigens.

## Conclusions

Current strategies for GBM therapy involve TMZ and radiation to induce tumor cell apoptosis, but tumors almost invariably recur and become resistant, suggesting they lack the direct cytotoxic potency to achieve tumor elimination. However, G207-NKG2D BiTE virus may complement this treatment strategy, as the sublethal pretreatment of GBM cells with radiation and TMZ may serve to further sensitize them to increased NKG2D BiTE activity. Furthermore, GSCs are a stubborn obstacle that must be overcome to achieve successful GBM therapy to prevent recurrence. While GSCs are non-permissive to the G207 virus, they remain targetable with the NKG2D BiTE. Therefore, the G207-NKG2D BiTE virus may serve as a gene therapy vector to achieve tumor-specific NKG2D BiTE expression which may then aid in the elimination of these GSCs.

10.1136/jitc-2023-008460.supp5 Supplementary data

## Data Availability

Data are available upon reasonable request.
