# Supplementary figures and images for: Targeting NKG2D ligands in glioblastoma with a bispecific T-cell engager is augmented with conventional therapy and enhances oncolytic virotherapy of glioma stem-like cells
Source: J Immunother Cancer. 2024 May 9;12(5):e008460. doi: 10.1136/jitc-2023-008460 (PMC11086472; doi:10.1136/jitc-2023-008460)

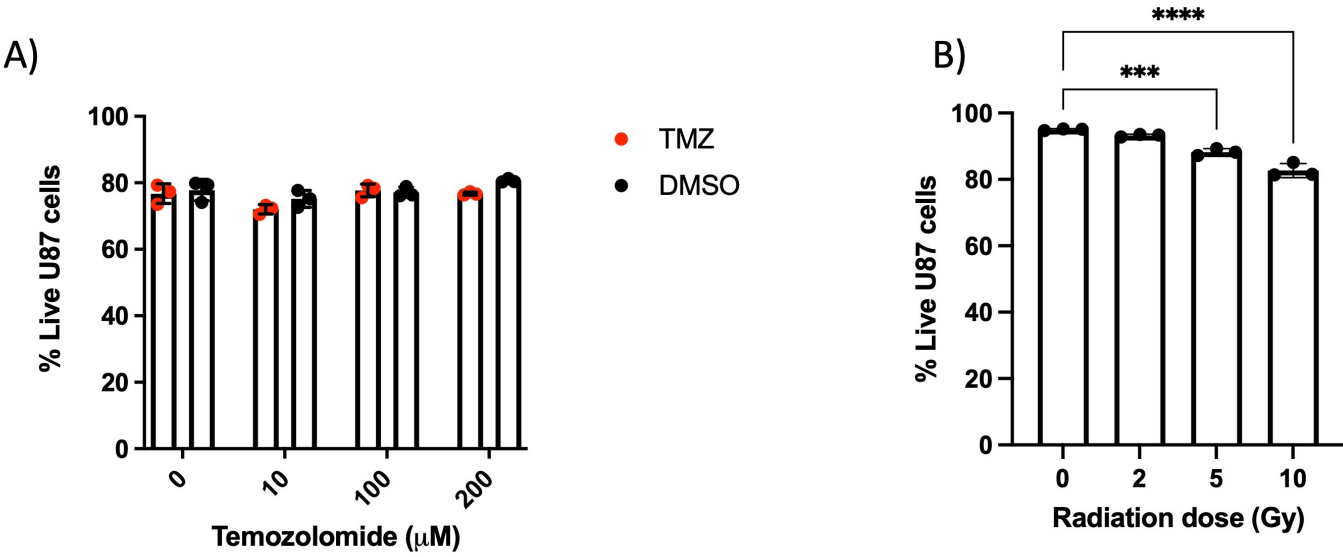

Supplement: Supplementary data [file jitc-2023-008460supp001.pdf]

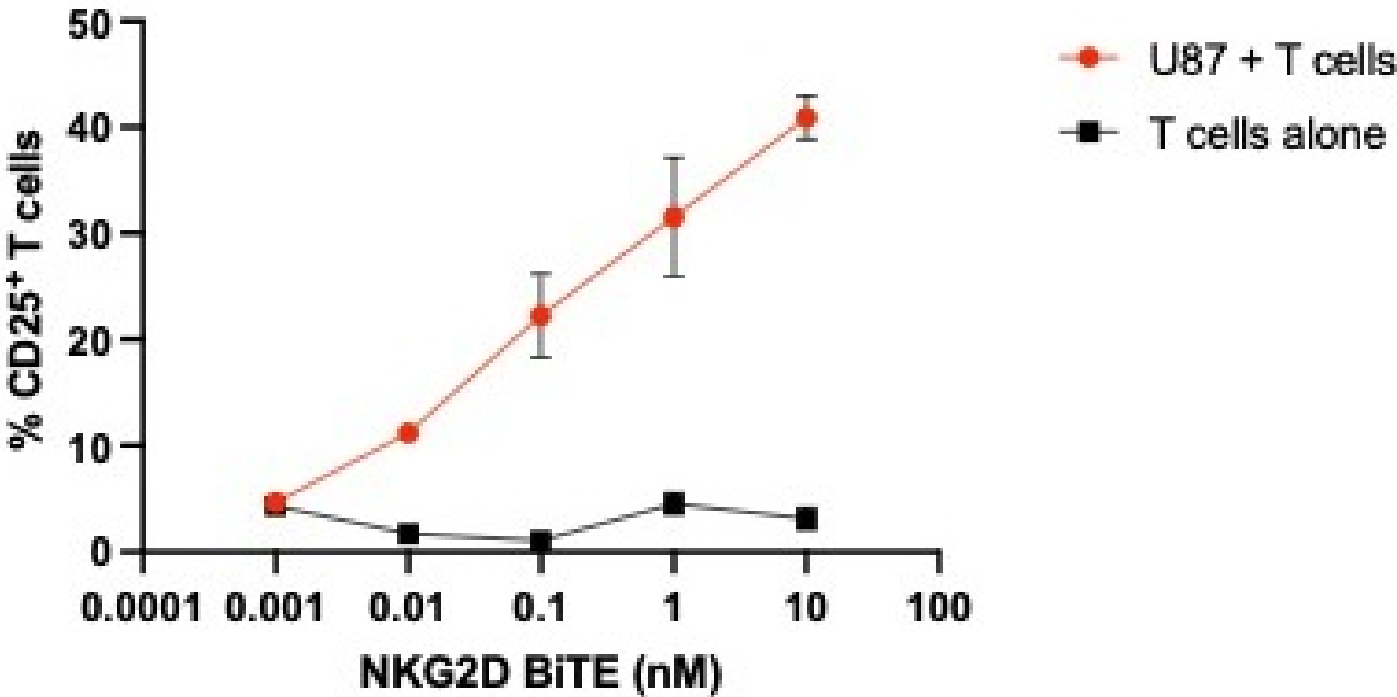

Supplement: Supplementary data [file jitc-2023-008460supp002.pdf]

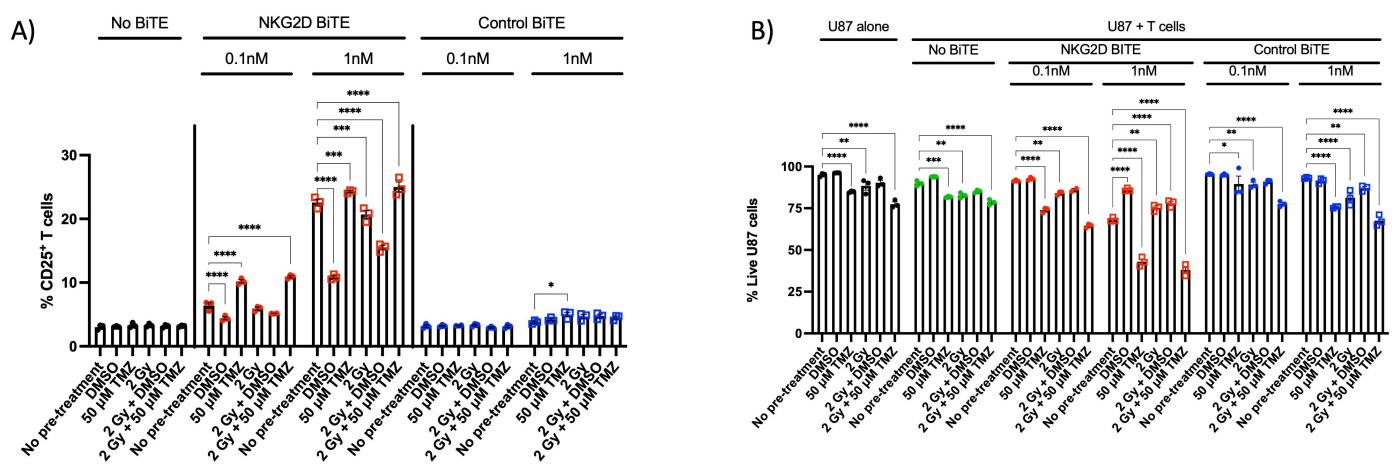

Supplement: Supplementary data [file jitc-2023-008460supp003.pdf]

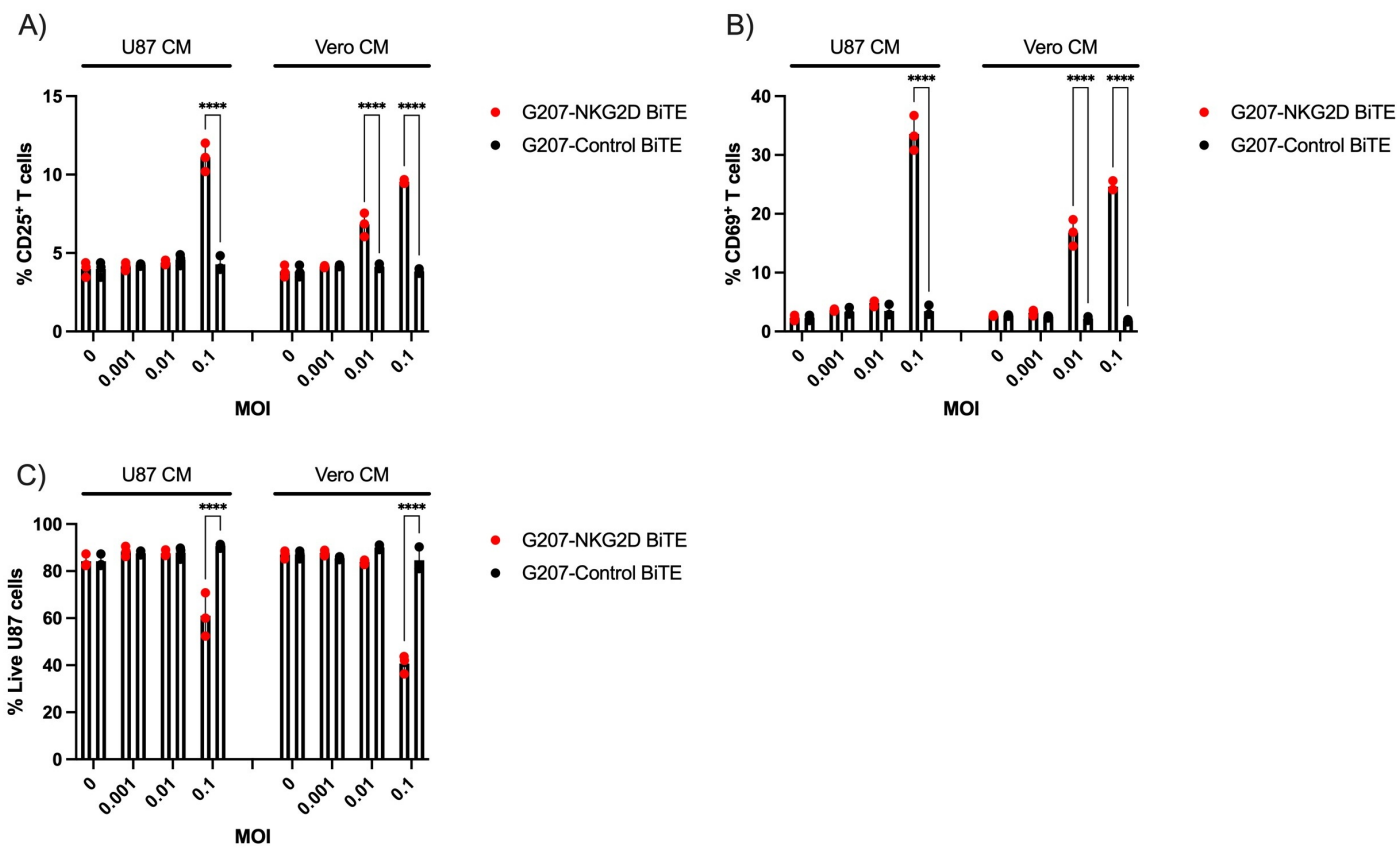

Supplement: Supplementary data [file jitc-2023-008460supp004.pdf]
