# Supplementary material for: Targeting NKG2D ligands in glioblastoma with a bispecific T-cell engager is augmented with conventional therapy and enhances oncolytic virotherapy of glioma stem-like cells
Source: J Immunother Cancer. 2024 May 9;12(5):e008460. doi: 10.1136/jitc-2023-008460 (PMC11086472; doi:10.1136/jitc-2023-008460)

Targeting NKG2D ligands in glioblastoma with a bispecific T cell engager is augmented with conventional therapy and enhances oncolytic virotherapy of glioma stem-like cells

Authors:
Richard Baugh, Hena Khalique, Emma Page, Janet Lei-Rossmann, Timothy Johanssen, Daniel Ebner, Olaf Ansorge, Len Seymour

Correspondence:
len.seymour@oncology.ox.ac.uk

Summary:
A Bispecific T cell engager (BiTE) targeting natural killer group 2 member D ligands (NKG2DLs) on glioblastoma (GBM) cells can activate T cells to direct cytotoxicity towards target GBM cells. Increased NKG2DL expression on GBM cells following radiation and temozolomide pre-treatment enhances NKG2D BiTE activity. 'Arming' the oncolytic herpes simplex virus (oHSV) G207 to secrete the NKG2D BiTE from infected cells enhances oncolytic virotherapy and directs cytotoxicity towards G207-resistant glioma stem-like cells (GSCs).

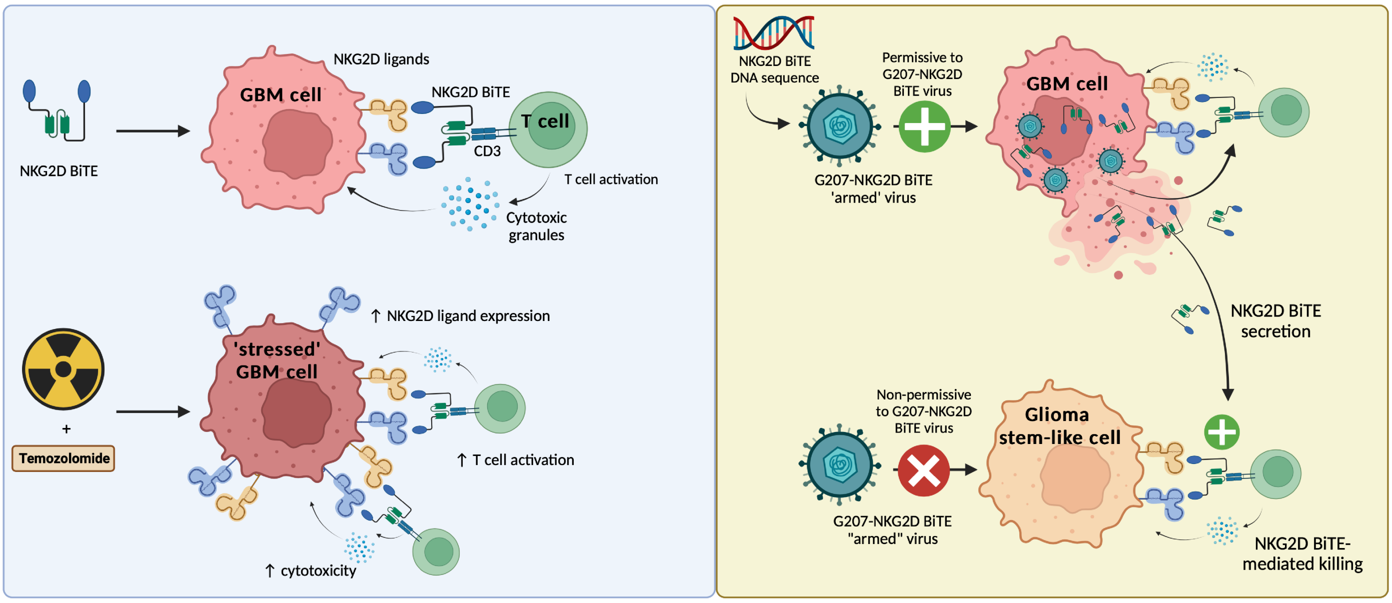

Supplement: Supplementary data [file jitc-2023-008460supp005.pdf]
